# Barriers and opportunities to implementation of sustainable e-Health programmes in Uganda: A literature review

**DOI:** 10.4102/phcfm.v9i1.1277

**Published:** 2017-05-29

**Authors:** Vincent M. Kiberu, Maurice Mars, Richard E. Scott

**Affiliations:** 1 Department of TeleHealth, Nelson R. Mandela School of Medicine, University of KwaZulu-Natal, South Africa; 2 Department of Community Health Sciences, University of Calgary, Canada; 3 Department of Family Medicine, University of Calgary, Canada

## Abstract

**Background:**

Most developing countries, including Uganda, have embraced the use of e-Health and m-Health applications as a means to improve primary healthcare delivery and public health for their populace. In Uganda, the growth in the information and communications technology industry has benefited the rural communities and also created opportunities for new innovations, and their application into healthcare has reported positive results, especially in the areas of disease control and prevention through disease surveillance. However, most are mere proof-of-concepts, only demonstrated in use within a small context and lack sustainability. This study reviews the literature to understand e-Health’s current implementation status within Uganda and documents the barriers and opportunities to sustainable e-Health intervention programmes in Uganda.

**Methods:**

A structured literature review of e-Health in Uganda was undertaken between May and December 2015 and was complemented with hand searching and a document review of grey literature in the form of policy documents and reports obtained online or from the Ministry of Health’s Resource Centre.

**Results:**

The searches identified a total of 293 resources of which 48 articles met the inclusion criteria of being in English and describing e-Health implementation in Uganda. These were included in the study and were examined in detail.

**Conclusion:**

Uganda has trialled several e-Health and m-Health solutions to address healthcare challenges. Most were donor funded, operated in silos and lacked sustainability. Various barriers have been identified. Evidence has shown that e-Health implementations in Uganda have lacked prior planning stages that the literature notes as essential, for example strategy and need readiness assessment. Future research should address these shortcomings prior to introduction of e-Health innovations.

## Introduction

The World Health Organization (WHO) defines e-Health as the use of information and communication technologies (ICTs) for health.^1^ e-Health enables public health and primary healthcare through activities such as disease surveillance, primary health data acquisition and analysis, support of community health workers, teleconsultation, tele-education, research and patient management. Managing a patient remotely using ICT is regarded as a more efficient means of delivering healthcare than transporting a patient from or a medical specialist to rural or remote locations.^2^ Learning from the developed world, sub-Saharan African countries are embracing e-Health as a means to improve accessibility to quality and equitable healthcare, especially for poor and vulnerable communities.^3,4^ These solutions use a variety of technological solutions, including online media, radio, fixed telephones, television and other devices for text messaging, teleconferencing, videoconferencing and sharing through e-mail.^5,6,7^ However, for most developing countries, e-Health remains a proof-of-concept activity, with only modest value demonstrated within small pilot projects.^3,6,8^

e-Health tools [mobile and fixed phones, voice over internet protocol, text and multimedia messaging] encourage communication between healthcare providers and their clients, sharing of information and knowledge among healthcare providers and establishing of better healthcare for patients.^9^ The use of the internet as a communication tool has also contributed to better disease management.^10,11^ Patients with chronic conditions are able to access treatment plans and individual medical records, consult with specialists at their convenience and access information on good healthy nutrition. Telemedicine services can potentially remedy some of the healthcare challenges in developing countries, especially in Africa, where distance, cost of equipment, time, limited human resources and lack of e-Health strategy remain major barriers that contribute to the poor quality of healthcare.^3,12,13^

While the potential of e-Health for sub-Saharan Africa is great, its uptake has been poor. A number of factors have been identified for this. These include: the excessive burden of disease in Africa^14^; the shortage of health professionals; a rapidly growing population which is outstripping the production of health workers; the low median age of people in sub-Saharan Africa of 19.5 years which, associated with poverty, results in low tax bases; unstable power provision; high telecommunication cost; lack of government will; and civil unrest which frequently results in damage to infrastructure. In contrast, opportunities exist as connectivity infrastructure (both internet and cellular phone based) grows and becomes cheaper, and widespread m-Health research in Africa affords the chance to leapfrog older, more expensive solutions. The literature on successful implementation of e-Health in Africa is limited to individual pilot or small-scale projects and is not generalisable.

Uganda is a landlocked country with a population of 41.2 million people, 85% of whom live in rural areas. The median age is 15 years. There are only eight physicians per 100 000 people who work in a pluralistic health system with a government-funded public sector, and private for profit and not for profit sectors supplemented by traditional and complementary medicine.

The public system is decentralised at district and sub-district levels ranging from national referral hospitals (NRHs), regional referral hospitals, general hospitals, health centres, to village health teams. The referral systems from lower to higher levels do not work well, due in part to poor transport and communication systems and staff shortages, with 44% of the established positions within the healthcare system vacant. The majority of the population is reliant on nurses, aid workers and traditional birth attendants for healthcare.^15^

The Government of Uganda recognises e-Health as an enabling platform to improve healthcare delivery by allowing doctors to consult and diagnose remotely, access patients’ medical information, provide district health information surveillance data and in addition facilitate research studies.^2,5^ The national data transmission backbone and e-Government infrastructure project (NBI/EGI) connect Uganda to neighbouring countries and links major towns, cities and government ministries and departments, with 48 government departments and six universities currently connected.

Internet penetration in Uganda is steadily growing – now estimated at 31%.^16^ Cellular phone coverage provided by private sector telecommunication companies has contributed to connectivity for the district, rural and remote areas. Improved internet bandwidth is seen as a major driver of voice and data communications, required for e-Health services.

Several studies conducted in developing countries, including Uganda, have demonstrated an increasing application of e-Health systems for healthcare delivery.^17^ The Government of Uganda recognises e-Health as a tool to improve health services delivery to its citizens but the country’s e-Health implementation status is unknown, and barriers and opportunities for sustainable e-Health implementation have not been documented. This study reviews the literature to understand the current status of e-Health implementation within Uganda and documents the barriers and opportunities to sustain e-Health intervention programmes in Uganda. This will inform policy- and decision-makers about critical areas of focus when designing and implementing sustainable e-Health innovations to strengthen healthcare delivery.

## Methods

A structured search of peer-reviewed literature on e-Health in Uganda was undertaken between May and December 2015, and complemented with hand searching and a document review of grey literature in the form of policy documents and reports online or from the Ministry of Health’s (MoH’s) Resource Centre.

The online databases, PubMed, Google Scholar, Scopus, CINAHL (Cumulative Index to Nursing and Allied Health Literature), Embase, EBSCO Health and Lilacs (Virtual Health Library) were searched. Published articles were complemented with a desk review of unpublished documents: reports and policies related to e-Health implementation in Uganda obtained from the MoH library. In PubMed, the following search string was used: ‘Uganda’ [Mesh] AND (‘Telemedicine’ [MeSH] OR ‘telemedicine’ [Text word] OR ‘telemedicine’ [All fields] OR ‘e-Health’ [All fields] OR ‘e-Health’ [Text word] OR ‘cell phones’ [Mesh] OR ‘Cell phones’ [All fields] OR ‘Electronic Medical Records’ [Mesh] OR ‘Electronic Medical Records’ [All fields]). For Google Scholar, the search was performed using four key phrases; e-Health in Uganda, telemedicine in Uganda, electronic medical records (EMRs) in Uganda and m-Health in Uganda (only the first 100 resources from each of the four Google Scholar searches were reviewed). These searches were supplemented by search of conference proceedings from ISTAfrica and Med-e-Tel [all conference years, in two thematic areas (e-Health and m-Health)], and hand searching of unpublished documents, including reports and policies obtained from the MoH Resource Centre. Titles and abstracts were reviewed to determine inclusion or exclusion. Inclusion criteria were broad: the document reported on e-Health activities in Uganda, and was in English.

## Results

The online searches identified 426 articles of which 146 articles were excluded as duplicates. In total, 293 unique resources were identified and reviewed: 280 from the database searches and 13 grey literature resources in the form of policy documents, reports and web articles. Inclusion criteria were not met by 245 articles and the remaining 48 articles were included in the study.

These 48 articles were classified according to the themes represented by the subject matter of the paper, thesis or report. The thematic areas were: m-Health – 23, electronic records systems – 8, telemedicine – 6, ICT – 6 and government policy documents and reports – 5 (Table 1). Most m-Health applications were proof-of-concept activities piloted as part of non-government organisation (NGO) projects and offered healthcare within particular communities in Uganda.

**TABLE 1:** A summary of articles and reports that met the inclusion criteria.

Articles, theses, and reports	Author name, year	Findings
**Telehealth**		
Into Africa: The telemedicine links between Canada, Kenya and Uganda.	House et al. (1987)	Study confirmed that new approaches to education and consultation can effectively be applied through ICT.
The Nakaseke multipurpose community telecentre in Uganda.	Mayanja (2001)	A telecentre was designed to provide a test bed for future investment in ICT for rural development.
Tele-dermatology web consultation and e-learning project Kampala-Mbarara, Uganda and Graz, Austria.	Kaddu (2007)	Despite being deemed successful, challenges encountered included technical and systems problems, plus economic and cultural limitations.
Factors affecting adoption, implementation and sustainability of telemedicine information systems in Uganda.	Isabalija et al. (2011)	Innovations around telemedicine are persistently being hindered by the lack of policy on use of telemedicine, insufficient knowledge and skills among health workers and resistance to change.
Feasibility and diagnostic accuracy of Internet-based dynamic telepathology between Uganda and Germany.	Wamala et al. (2011)	Internet-based conferencing systems offer an inexpensive method of obtaining a primary diagnosis by telepathology and consulting on cases that require subspecialty expertise.
A framework for designing sustainable telemedicine information systems in developing countries.	Kituyi et al. (2012)	The key requirements for designing sustainable telemedicine information systems in developing countries were identified as the need for speed, ease of use and affordability.
**Electronic medical records**		
Using HMIS for monitoring and planning: The experience of Uganda Catholic Medical Bureau.	Mandelli and Giusti (2005)	The existing HMIS can be used as a tool to monitor the effects of managerial decisions.
Creation and evaluation of EMR-based paper clinical summaries to support HIV-care in Uganda, Africa.	Were et al. (2010)	By taking advantage of data stored in EMRs, efficiency and quality of care can be improved through clinical summaries, even in settings with limited resources.
Use of an innovative, affordable, and open-source short message service-based tool to monitor malaria in remote areas of Uganda.	Asiimwe et al. (2011)	Use of SMS-based reporting systems have the potential to improve timeliness in reporting of specific, time-sensitive metrics at modest cost, while bypassing current bottlenecks in the flow of data.
Electronic medical records and same day patient tracing improves clinic efficiency and adherence to appointments in a community-based HIV/AIDS care programme, in Uganda.	Alamo et al. (2012)	EMR and same day patient tracing can significantly reduce missed appointments (LTFU) and improve clinic efficiency.
Review of developing country health information systems.	Foster (2012)	Uganda has strong evidence of planning and implementation of adequate ICT infrastructure for health facilities.
A study of the preconditions for a sustainable implementation of a digital health system in Uganda.	Gårdstedt et. al. (2013)	There is no need for sophisticated technologies for Uganda to achieve sustainable ICT projects within healthcare.
e-Health at outpatient clinics in Uganda.	Hindemark (2013)	Because of the multitude of non-compatible e-Health projects in Uganda, the report calls for a consolidation of efforts and sharing of information among the e-Health application developers of Uganda.
Designing an architecture for secure sharing of personal health records – A case of developing countries.	Ssembatya (2014)	Identity-based encryption can be extended to mobile phones to secure patient health records beyond the hospital server domain.
**m-Health**		
Responding to the human resource crisis: Peer health workers, mobile phones, and HIV care in Rakai, Uganda. AIDS Patient Care.	Chang et al. (2008)	A simple and inexpensive clinical and adherence monitoring intervention leveraging PHWs empowered with mobile phones can successfully be implemented in a rural resource limited setting.
m-Health: Mobile phones in HIV prevention in Uganda.	Salomonsson (2010)	Mobile phones can be both feasible and effective in HIV prevention campaigns in east African settings despite structural constraints such as lack of electrical power and illiteracy.
Using mobile phones to improve clinic attendance amongst an antiretroviral treatment cohort in rural Uganda: A cross-sectional and prospective study.	Kunutsor et al.(2010)	Mobile phones have a potential for use in resource-constrained settings to substantially improve the clinical management of HIV/AIDs.
Using SMS for HIV/AIDS education and to expand the use of HIV testing and counselling services at the AIDS Information Centre (AIC) Uganda.	Hoefman and Apunyu (2010)	Study outcome saw a high acceptance rate of the SMS survey and increase in the number of people accessing HCT.
Using personal digital assistants to improve healthcare delivery in Uganda (Doctoral dissertation, Malmo University, Sweden).	Kirunda (2010)	The use of PDAs can improve healthcare delivery in rural health facilities.
Impact of an m-Health intervention for peer health workers on AIDS care in rural Uganda: A mixed methods evaluation of a cluster-randomised trial.	Chang et al. (2011)	Interventions for m-Health registered improvements in patient care, logistics and broad support among patients, clinic staff and PHWs.
Cell phone usage among adolescents in Uganda: Acceptability for relaying health information.	Mitchell et al. (2011)	Need for effective HIV prevention programmes that can reach large audiences at low cost and are culturally relevant for the Ugandan context.
You have an important message! Evaluating the effectiveness of a text message HIV/AIDS campaign in Northwest Uganda.	Chib et al. (2012)	Potential of m-Health tools when extended to millions of mobile phone users as part of an integrated health campaign approach.
Mobile phones in health care in Uganda: The Applab study.	Nchise et al. (2012)	A need to integrate a referral system to registered health professionals and their facilities, and the need for education and/or marketing strategy with an indigenous branding to address the misconception of the brand name ‘Google SMS’.
m-Health and developing countries: A successful obstetric care model in Uganda.	DeStigter (2012)	Uganda still experiences challenges due to expensive equipment and high electrical power requirements hindering developments in e-Health.
Usability testing of a prototype phone oximeter with healthcare providers in high- and low-medical resource environments.	Hudson et al. (2012)	User feedback was positive and the overall usability was high.
High acceptability for cell phone text messages to improve communication of laboratory results with HIV-infected patients in rural Uganda: A cross-sectional survey study.	Siedner et al. (2012)	Cell phone text messaging for communication of abnormal laboratory results is highly acceptable in a cohort of HIV-infected patients in rural Uganda.
Optimising network connectivity for mobile health technologies in sub-Saharan Africa.	Siedner et al. (2012)	Addition of SMS to standard GPRS cellular network connectivity can significantly reduce network connection failures for mobile health applications in remote areas.
Perceptions and acceptability of m-Health interventions for improving patient care at a community-based HIV/AIDS clinic in Uganda: A mixed methods study.	Chang et al. (2013)	Study findings were vital to guide future design and implementation of m-Health interventions in this setting, optimising their chances for success.
Cost analyses of PHW and m-Health support interventions for improving AIDS care in Rakai, Uganda.	Chang et al. (2013)	The PHW intervention (m-Health) was found to be cost saving.
Vulnerabilities in m-Health implementation: A Ugandan HIV/AIDS SMS campaign.	Chib et al. (2013)	Interactive SMS quiz design motivated recipients with the correct HIV/AIDS knowledge to respond (and thus become eligible for free HIV screening).
The role of m-Health in Uganda: A tool to reach development.	Mattsson and Sabuni (2013)	ICT tools such as m-Health were identified. However, collaboration between NGOs and government must exist to build communication and organisational structure.
Response patterns to interactive SMS health education quizzes at two sites in Uganda: A cohort study.	Lepper et al. (2013)	Future research should focus on developing evidence-based guidelines for the design, implementation and evaluation of SMS-based interventions.
The role of mobile health technologies in improving community health seeking practices in rural Uganda.	Liu and Lin (2014)	Study showed a critical gap in the local healthcare infrastructure that needs to be addressed in order to establish more efficient delivery of healthcare services.
A pilot study on mobile phones as a means to access maternal health education in eastern rural Uganda.	Roberts et al. (2014)	Providing local communities with mobile maternal health education offers a new potential method of reducing maternal mortality.
Demographic and psychosocial characteristics of mobile phone ownership and usage among youth living in the slums of Kampala, Uganda.	Swahn et al. (2014)	Given that nearly half of the youth own and use phones daily, new research is needed to determine next steps for mobile health (m-Health).
A model of e-Health acceptance and usage in Uganda: The perspective of online social networks.	Miiro and Maiga (2014)	A generic social networked model was developed that can be adopted for use by other transitioning countries.
Introduction of mobile phones for use by volunteer community health workers in support of integrated Community Case Management (iCCM) in Bushenyi District, Uganda: Development and implementation process.	Tumusiime et al. (2014)	Robust mobile phone–based system may contribute to efficient delivery of iCCM by trained volunteer CHWs in rural settings in Uganda.
**Information and communication technologies**		
Application of information and communication technology (ICT) in health information access and dissemination in Uganda.	Omona and Ikoja-Odongo (2006)	All stakeholders in the health sector need to support and promote ICT as the most effective tool for health information access and dissemination. In addition, a number of challenges must be addressed if full benefit of the use and application of ICT is to be realised in Uganda.
ICTs and health in Uganda: Benefits, challenges and contradictions.	Litho (2007)	Development in ICTs for health creates opportunities and benefits for Uganda to step up and embrace sophisticated modes of healthcare delivery.
Information and communication technology and community-based health sciences training in Uganda: Perceptions and experiences of educators and students.	Chang et al. (2012)	Internet access in rural educational sites is still lacking, but students and educators appear eager to utilise this resource if availability improves.
A framework for sustainable implementation of e-medicine in transitioning countries.	Isabalija et al. (2013)	e-Medicine sustainability in sub-Saharan Africa is affected by institutional factors.
Understanding ICT behaviours among health workers in sub-Saharan Africa: A cross-sectional study for laboratory persons in Uganda.	Kasusse et al. (2014)	It is viable/feasible to pilot informatics projects as strategies to build bridges and develop skills for an e-Health landscape in laboratory services with a bigger financial muscle.
A structured approach for evaluating ICT contributions to development. PhD Thesis. Stockholm University, Faculty of Social Sciences, Department of Computer and Systems Sciences. Makerere University, Uganda.	Kivunike (2015)	Proposes a model and criteria for the evaluation of ICT-related development, recommends complementary qualitative and quantitative approaches and proposes indicators to appropriately evaluate the ICT contribution to development.
**Government policy documents and reports**		
National Health Policy: Reducing poverty through promoting people’s health.	Ministry of Health-Uganda (2009)	Government of Uganda official policy document.
Health Sector Strategic Investment Plan III.	Ministry of Health-Uganda, (2010)	The health sector strategic plan three for the period 2010/11–2014/15.
The Second National Health Policy: Promoting people’s health to enhance social-economic development.	Ministry of Health-Uganda, (2010)	Government policy document about the health sector in Uganda.
Computerised and integrated human resource information system.	Ministry of Health-Uganda, (2011)	Government policy document about human resources in health.
Uganda Health System Assessment.	Ministry of Health-Uganda (2011)	A report on the five health systems building blocks of the healthcare system in Uganda.

CHWs, community healthcare workers; EMRs, electronic medical records; HCT, HIV counselling and testing; HMIS, Health Management Information System; LTFU, loss to follow-up; NGO, non-government organisation; PDA, personal digital assistants; PHWs, peer health workers.

## Discussion

e-Health in Uganda started in the 1980s when an audio satellite connection was established between Uganda and Canada for postgraduate education support, to which was added electroencephalogram (EEG) transmission.^18^ The next phase was computerisation of the national databank at the MoH using Microsoft Access (1997–2001) followed by EpiInfo in 2002 for district health information analysis.^63^ In 2011, the MoH adopted and rolled out the District Health Management Information System (DHIS2) to the 112 districts of Uganda.^64^ This electronic system, which is widely used in Africa, aims to strengthen routine health data reporting from the district level to the national headquarters level (MoH), replacing the existing paper-based system.

Uganda has a fast growing ICT industry, especially in mobile technology. This has enabled innovations around m-Health tools, for instance, the ICT4MPOWER project initiated by the MoH, Uganda Communications Commission (UCC) and the Ministry of ICT (MoICT) aimed at strengthening the flow of information right from community to national levels of the healthcare system. The first site to benefit was Mukono Health Centre IV.^6,57^ In 2011, students of Makerere University developed ‘WinSenga’ – a foetal heart rate monitor using a smart phone.^65,66^ Another mobile application ‘Matibabu’ was developed in 2012 to perform a non-invasive malaria test, obviating the need to visit a laboratory technician at a health facility to draw a blood sample.^67^

An EMR system was implemented to improve access to antiretroviral treatment at the Reach out Mbuya HIV/AIDs clinic, which reduced missed appointments and improved clinic efficiency.^27^ In a similar setting, an m-Health intervention was introduced for community-based peer health workers (PHWs) providing HIV/AIDs care at the Rakai Service Health Program in rural Uganda. The PHWs were satisfied with the system which was found to improve communication among PHWs, patients and staff members.^35^ Text to Change, in partnership with AIDS Information Centre and Celtel mobile network, piloted an SMS mobile phone–based platform to scale up HIV/AIDs awareness and encourage participants to access HIV counselling and testing (HCT).^33,68^ The system created awareness through disseminating preventive and general health-related information to complement existing campaigns on prevention of HIV transmission and voluntary HCT.

The Uganda National Drug Authority developed an SMS-based platform (U-reporting) that is used to generate the national procurement and supply management plan.^69^ Similarly, in December 2011, the MoH-Uganda launched and successfully implemented mTrac (Mobile Tracking) as a RapidSMS-based health management information tool designed to strengthen health systems in Uganda using a basic mobile phone. The goal was to speed up response time and accountability while reporting on disease surveillance and medicine tracking in all 5000 health facilities in Uganda,^70^ which to a great extent has been achieved. Mobile phones have further been utilised to enhance access to health information and cell phone–based surveys. The MoH adopted and installed a knowledge management portal and a digital library, then a computerised and integrated human resource information system with several modules, a human resources (HR) management system, a licensure tracking system and a training and certification module.^61^

Uganda’s healthcare system has benefited from donor funding. The Canadian International Development Research Centre funded a telemedicine project based on the East African Telemedicine Project of the 1980s, ‘HealthNet’. The aim was to improve distance education among medical students and to enable resource sharing at Mulago hospital with other health workers in remote areas. In addition, a pilot project strove to enhance data capture, storage, interpretation and retrieval of patients’ information among health workers using a handheld computer (portable digital assistant).^71^

Ugandans seek healthcare through a range of telehealth or m-Health-related means. This includes calling into the local radio medical talk show to get medical advice from the doctor, while those with internet access probably subscribe to daily health tips via their mobile phones and e-mail addresses. In addition, they also use the phone application to diagnose their health conditions, search the internet for health information, post queries for alternative opinions and share experiences on health and illnesses on social networks.^72^

In June 2014, the Government of Uganda commissioned a new building complex for Masaka Referral Hospital that will be fitted with telemedicine related equipment such as televisions/monitors, internet connections, cameras and many others, which will allow patients to link with medical consultants at the NRH – Mulago, minimising physical daily referrals. Similarly, the University of Virginia (USA) and the Mbarara University of Science and Technology link by videoconferencing for interactions in research, education and clinical practices among students at both universities.^73^

### Status of e-Health programmes in Uganda

The literature identified a spectrum of barriers and opportunities relevant to Uganda. These are discussed below according to the themes previously identified in Table 1.

According to Uganda’s 2013 national e-Health policy,^5^ most e-Health applications and products have been run in silos and are not interoperable or compatible, preventing sharing of information and services. Several technology innovations have remained as pilots for life as they are not interoperable as a result of divergent platforms. As in other developing countries, such e-Health initiatives are donor funded and often remain a proof-of-concept wherein technology is demonstrated within a limited context.^5,6^ The majority of such initiatives tend to remain as small or medium-sized ICT projects or have stalled or stagnated with the stoppage of donor funding. This has been attributed to lack of local ownership and accountability, support and funding.^6^ Poor coordination and communication, and a lack of proper e-Health implementation frameworks, are also cited as major challenges to sustainable e-Health programmes.^55^

Uganda’s National e-Health Policy also identifies the non-existence of e-Health standards and systems as challenges.^5^ There are no national guidelines for secure management of individuals’ electronic health information and services, placing personal data at risk. This may eventually be a barrier to adoption of e-Health and the realisation of its benefits such as enhancement of health information sharing and effective management of the health system. The Uganda Medical and Dental Practitioners Council, with an oversight function for legal and regulatory compliance, lacks competence in the area of e-Health and has not provided ethical guidelines.^74^

e-Health innovations can reduce healthcare costs and enable access to better quality healthcare, provided there is adequate infrastructure. However, consistent power blackouts, loss of internet connectivity and the presence of an unskilled health workforce hinder its uptake.^64^ Despite the existing barriers, sustainable e-Health programmes can be implemented in Uganda. Similar to other African countries, Uganda has implemented 3G and 4G broadband internet services to raise internet penetration.^16,75^ However, the experience is that 3G and 4G services are not available in most rural areas as the people are too poor to make it economically viable.^16^

### Telehealth systems

In the 1980s, the first teleconferencing link established between University of Nairobi, Makerere University and their Canadian counterparts failed because of political turbulence. Meanwhile, the programme provided for personal physician collaboration where faculty from Uganda and Kenya received training in particular fields, while those from Canada served as visiting faculty at the different universities.^18^ In addition, international and local partners together with UNESCO funded a three-year telecentre project at Uganda’s Nakaseke Multipurpose Community Telecentre (MCT) to provide services such as printing, internet/emails and telephone services. In addition, telemedicine services were offered between Nakaseke hospital and Mulago national referral hospital, aimed at improving quality of life for the community. Similar to the internet-based telepathology link between Uganda and Germany that was faced with slow internet speed,^22^ the MCT project was constrained by poor telecommunications infrastructure, persistent power blackouts and an illiterate community.^19^

Lack of knowledge and skills about telehealth, and the absence of policy and guidelines for the use of telehealth at hospitals, have been cited as major barriers to its adoption in Uganda.^21^ Consequently, studies have recommended designing a suitable and appropriate telemedicine framework that would lead towards adoption of sustainable telemedicine programmes in developing countries like Uganda.^21,23^ The Uganda–Austria Tele-Dermatology Web Consultation and E-Learning Project was successfully implemented. However, that project faced barriers such as consistent interruption with the internet connections, lack of medical specialists (notably dermatologists), limited support from the ICT technical staff and most importantly resistance from the local dermatologists because they looked at the process of transmitting images as an extra workload.^20^

### Information and communication technology knowledgeable workforce

Human resources for e-Health comprise health workers, ICT professionals and electronic content developers. These health cadres have low levels of computer literacy and skills to use ICT equipment and systems, especially those in rural areas.^56^ Integrating ICT within the current hospital setting is seen as an extra burden to the nurses and doctors, an added responsibility which draws them away from their core duties. Further, in some health facilities where health workers are computer literate, computers are not used for routine official work. There is also a shortage of qualified ICT personnel to manage and maintain technology equipment and to support health workers to use such equipment and systems, especially at lower health facilities.^55^

Countering this reality, ICT development has had a profound impact on Uganda’s economy.^28^ Uganda’s Vision 2040 puts in focus the need for developing ICT infrastructure in education and other government structures to take advantage of the ICT-enabled environment, and to prepare a future generation of ICT-proficient citizens. The rapid development in ICT and its use in many of the public sectors in Uganda present an opportunity for collaboration and partnership for health information access and dissemination, timely access to healthcare delivery and to enhance the quality of teaching and learning. However, in order for the health sector to tap into the benefits of e-Health, organisations need to be assessed for readiness and the right policies, strategies, regulations and resources need to be in place. Such policies and strategies might include the National e-Health Strategy, Health Sector Strategic Investment Plan and Health Systems Strategic Report.

### m-Health

In addition, there is promise for m-Health interventions and increased use of mobile phone technology in Uganda in both rural and urban communities despite the poverty levels.^44,50^ The evolution of the cell phone into the multi-featured smartphone makes them handy and sophisticated devices to carry, and allows transfer of high resolution images. These features can facilitate health education, and store and forward telemedicine and disease prevention initiatives.^36^

Uganda has adopted e-Health services such as m-Health programmes to improve healthcare delivery.^6^ However, most of such innovations are proof-of-concept, donor funded and lack sustainability and scalability plans.^6,8^ Mobile phones are very flexible and cost-efficient devices for data acquisition and storage. To that effect, a secure architecture for handling personal health records on mobile phones was proposed.^29^ The assumption was that the cheaper bandwidth would lead to high-speed internet to support technology-enabled services. Health institutions, medical practitioners and other citizens – regardless of geographical location – would be able to link with and share or seek medical expertise and knowledge. For instance, a doctor in one part of the country could conduct a consultation in another part of the country via video conferencing, while health workers could acquire more knowledge and skills using tele-education and teleconsultation.

### Electronic medical record systems

Electronic medical records (EMRs) are used for storage, management and retrieval of patients’ data.^25^ Shuaib et al. specifically reported that developed countries are doing well as far as patients’ data are concerned, unlike their counterparts in developing countries that still experience some challenges,^76^ particularly with ethical issues in accessing patients’ data.^77,78^ For instance in Kenya, despite the rollout of EMRs to several sites, a project was hampered by limitations such as inadequate infrastructure, system interoperability and lack of skilled expertise.^79^ Similar challenges coupled with inadequate funding and resistance from health workers were encountered in Uganda during implementation of EMRs, such as OpenMRS, mTrack and DHIS2.^64,70^

### Keys to sustainable e-Health programmes

Regardless of the presence of a strategy to guide implementation, or an evidence based need, if the setting is not ‘ready’ to use these innovations, they will not succeed.^80^ e-Health readiness assessment in relation to physical infrastructure, technology equipment, user and managers’ skills, policies, regulations and guidelines should be undertaken prior to implementing any e-Health system.^81,82^ Three factors – all part of readiness – have been identified as hindrances to adopting telemedicine in Uganda: lack of knowledge and skills, lack of policy and resistance from healthcare workers.^21^ There is need for evidence of the impact of, and readiness for, e-Health systems before further investment of resources in development and implementation of such systems.^17^ Failure of e-Health projects has been attributed to poor initial planning and research design, insufficient computing skills, lack of change management and lack of technology readiness.^83^ It has been recommended that policy and healthcare managers undertake adequate planning and make better use of their resources for successful and sustainable e-Health projects.^84^ There is no evidence in the literature of e-Health readiness assessment having been conducted prior to implementation of any e-Health projects in Uganda. Studies have shown how a few developing countries (Ethiopia, South Africa and India)^3,12,85,86^ are successfully adopting e-Health systems.^87,88,89^ Developing countries like Uganda should learn from the experiences of such countries to innovate and implement their own sustainable e-Health programmes.

Opportunities exist to respond to many of the challenges identified. Conventional guidance highlights the requirement for a defensible (health needs based, evidence based and prioritised) e-Health strategy that invokes e-Health only when demonstrated to offer viable solutions.^90,91^ Similarly, the literature shows sustainability to be enhanced by following a traditional process extending from needs and readiness assessment, through piloting and change management, to evaluation, although the rise of ‘spontaneous telemedicine’ offers an alternative route particularly for developing countries.^92^ While response to clear need for a change is emphasised as the stimulus for readiness to change, it must be multilateral considering the perspective and needs of the many stakeholders involved, including providers, patients, payers, etc.^95^ Introducing solutions that do not increase the workload of providers or inconvenience for patients, and consideration of transfer of skills are also necessary. Finally, construction of an enabling legal and ethical policy environment, and preparation of clear best practice guidelines have also been identified as easing implementation.^91^

### Significance to primary health care

e-Health is among the building blocks upon which modern health sectors are built. This involves combined use in the health sector of ICTs for learning, research, data acquisition, surveillance, storage and access to patient data and clinical care even at a distance. In developing countries, e-Health solutions have the potential to improve health through enhancing capacity of the health workforce, especially where traditional means are lacking. They may also improve access to relevant information through eliminating constraints like distance and time in accessing information and sharing knowledge.

## Conclusion

The literature shows a plethora of e-Health-related activities within Uganda over many years. Uganda has trialled several e-Health and m-Health solutions but most were donor funded, operated in silos and lacked sustainability. Evidence has shown that e-Health implementations in Uganda have lacked prior planning stages of need and readiness assessment, noted in the literature as essential. It is clear that the healthcare system in Uganda has taken the initiative to adopt e-Health programmes such as mobile applications and health information systems. Several barriers have been identified. Furthermore, several ICT infrastructural enhancements geared towards reducing costs of internet bandwidth will contribute to future implementation of more e-Health programmes, enabling teleconsultations, tele-education and teleconferencing activities. However, implementation must lead to sustained programmes. This requires evidence based and defensible strategy, needs based applications, a skilled and a knowledgeable workforce and a ‘ready’ setting. There is no evidence in the literature of a holistic ‘e-Health Readiness Assessment’ having taken place in any of the existing projects prior to implementation. Further research on the development and use of e-Health readiness tools relevant to Uganda is required, and awareness of the need to conduct e-Health readiness assessment during the planning of e-Health programmes needs to be raised.
